# Celecoxib-Loaded Cubosomal Nanoparticles as a Therapeutic Approach for *Staphylococcus aureus* In Vivo Infection

**DOI:** 10.3390/microorganisms11092247

**Published:** 2023-09-06

**Authors:** Samar Zuhair Alshawwa, Thanaa A. El-Masry, Mohamed Nasr, Ahmed Y. Kira, Hadil Faris Alotaibi, Al-Sayed Sallam, Engy Elekhnawy

**Affiliations:** 1 Department of Pharmaceutical Sciences, College of Pharmacy, Princess Nourah bint Abdulrahman University, P.O. Box 84428, Riyadh 11671, Saudi Arabia; 2 Pharmacology and Toxicology Department, Faculty of Pharmacy, Tanta University, Tanta 31527, Egypt; 3 Department of Pharmaceutics and Industrial Pharmacy, Faculty of Pharmacy, Helwan University, Cairo 11790, Egypt; 4 Department of Pharmaceutics, Faculty of Pharmacy, Delta University for Science and Technology, Mansoura 11152, Egypt; 5 Al-Taqaddom Pharmaceutical Industries, Amman 11947, Jordan; 6 Pharmaceutical Microbiology Department, Faculty of Pharmacy, Tanta University, Tanta 31527, Egypt

**Keywords:** bacterial infection, repurposing, inflammatory markers, histological features, nanostructures, oral delivery system

## Abstract

There is a great need for novel approaches to treating bacterial infections, due to the vast dissemination of resistance among pathogenic bacteria. *Staphylococcus aureus* are ubiquitous Gram-positive pathogenic bacteria and are rapidly acquiring antibiotic resistance. Here, celecoxib was encapsulated into cubosomal nanoparticles, and the particle morphology, size distribution, zeta potential, entrapment efficiency, and celecoxib release were evaluated in vitro. Also, a systemic infection model in mice elucidated the in vivo antibacterial action of the celecoxib cubosomes. Cubosomes are a nanotechnology-based delivery system which can adhere to the external peptidoglycan layers of Gram-positive bacteria and penetrate them. The size distribution investigation revealed that the prepared celecoxib-loaded cubosomes had a mean particle size of 128.15 ± 3.04 nm with a low polydispersity index of 0.235 ± 0.023. The zeta potential measurement showed that the prepared cubosomes had a negative surface charge of −17.50 ± 0.45, indicating a highly stable nanodispersion formation with little susceptibility to particle aggregation. The cubosomal dispersion exhibited an entrapment efficiency of 88.57 ± 2.36%. The transmission electron micrograph for the prepared celecoxib-loaded cubosomes showed a narrow size distribution for the cubosomal nanoparticles, which had a spherical shape and were non-aggregated. The tested cubosomes diminished the inflammation in the treated mice’s liver and spleen tissues, as revealed by hematoxylin and eosin stain and Masson’s trichrome stain. The immunostained tissues with nuclear factor kappa B and caspase-3 monoclonal antibodies revealed a marked decrease in these markers in the celecoxib-treated group, as it resulted in negative or weak immunostaining in liver and spleen that ranged from 4.54% to 17.43%. This indicates their inhibitory effect on the inflammatory pathway and apoptosis, respectively. Furthermore, they reduced the bacterial burden in the studied tissues. This is alongside a decrease in the inflammatory markers (interleukin-1 beta, interleukin-6, cyclooxygenase-2, and tumor necrosis factor-alpha) determined by ELISA and qRT-PCR. The IL-1β levels were 16.66 ± 0.5 pg/mg and 17 ± 0.9 pg/mg in liver and spleen, respectively. Also, IL-6 levels were 85 ± 3.2 pg/mg and 84 ± 2.4 pg/mg in liver and spleen, respectively. In conclusion, the current study introduced cubosomes as an approach for the formulation of celecoxib to enhance its in vivo antibacterial action by improving its oral bioavailability.

## 1. Introduction

*Staphylococcus aureus* is a Gram-positive bacterium that belongs to the normal flora. It is an opportunistic pathogen that can result in various illnesses ranging from mild skin infections to life-threatening systemic ailments [1]. These bacteria are rapidly acquiring resistance due to the misuse of antimicrobials in different regions, especially in developing countries [2,3]. In addition, they possess numerous virulence factors like the production of various enzymes and toxins and biofilm formation [4]. Thus, these problematic bacteria have attracted scientists’ attention, and multiple studies have been performed to elucidate novel alternatives to current antibiotics to combat such pathogenic bacteria [4,5,6].

Drug repurposing or drug repositioning means the identification of novel therapeutic activities from the currently existing FDA-approved drug molecules that are used clinically. It is a promising approach for treating various human ailments like bacterial infections, as the currently approved drugs used to treat different diseases are investigated for their possible antibacterial activity [7,8]. This approach benefits from the fact that FDA-approved drugs’ pharmacological and toxicological characteristics are already known. It is well-known that drug discovery is a laborious, expensive, time-consuming, and highly risky procedure. Thus, the novel tactic of drug repurposing is used to increase the rate of success of drug development [9]. This strategy is more advantageous than the traditional approach of the drug discovery process owing to a reduction in the duration of drug development, low cost, and minimum risk of failure.

Non-steroidal anti-inflammatory drugs (NSAIDs) are among the most commonly prescribed classes of drugs. They possess long-standing and wide-ranging therapeutic applications, from the salicylate-containing willow leaves to the recent highly selective cyclooxygenase-2 (COX-2) inhibitors. Celecoxib is one of the non-steroidal anti-inflammatory compounds that are widely employed to treat symptoms of inflammation like arthritis [10].

Celecoxib is a poorly water-soluble, very lipophilic drug that has a poor oral bioavailability ranging between 22% and 40% when administered in the form of traditional capsules [11]. It shows no signs of uneven distribution in vivo and has a volume of distribution in humans of 455 L [12]. The lipophilic nature of celecoxib may be responsible for its higher volume of distribution and its low water solubility, both indicative of low bioavailability. Celecoxib undergoes substantial human metabolism and is eliminated predominantly through metabolites [11]. Celecoxib’s bioavailability has been improved through the use of a variety of formulation strategies, including a self-micro-emulsifying delivery system [13], solid lipid nanoparticles (NPs) [14], the formation of a complex with β-cyclodextrins [15,16], the formation of eutectic mixtures [17], and the utilization of silica–lipid hybrid microcapsules [18].

With the growing concern about antimicrobial resistance and the need for novel therapeutic strategies, repurposing existing drugs has emerged as a promising approach. Celecoxib has recently gained attention for its potential antibacterial activity [19,20]. However, it is essential to note that all previous studies investigating its antibacterial properties have been conducted using traditional delivery systems. In our research, we aim to enhance the antibacterial activity of celecoxib by utilizing cubosomal nanoformulations.

The development of nanoformulations has opened new avenues for enhancing the antibacterial activity of different antibiotics, including cephradine [21,22], cefadroxil [23], and azithromycin [24]. There are different approaches for the delivery of poorly water-soluble drugs like coacervates which are condensed, liquid-like droplets, usually formed with oppositely charged polymeric molecules [25]. Also, cubosomes are a nanotechnology-based delivery system and are biocompatible carriers in drug delivery applications. They are made of particular amphiphilic substances in varying proportions. Cubosomes are bicontinuous cubic phases that have distinctive physicochemical properties [26]. These systems can include a wide range of hydrophobic, hydrophilic, and amphiphilic drugs with enhanced bioavailability; accordingly, they are an interesting area of research [27]. They can potentially increase the absorption of the drug and extend its release owing to their lipid bilayer, which is structurally similar to the lipid bilayer of biological membranes. Additionally, their biocompatibility and bioadhesion make them attractive as a probable way to improve the oral delivery of drugs [28,29,30,31].

Cubosomes can cling to the external peptidoglycan layers of Gram-positive bacteria and then penetrate the bacterial cell. The contact in Gram-negative bacteria occurs in a two-stage process involving fusion with the outer lipid membrane and subsequent diffusion through the inner wall. The inherent self-assembly property of cubosomes confers upon them a unique potential to facilitate the transportation of payloads via the process of membrane fusion [32]. The fusion absorption method described facilitates the effective and expeditious internalization of cubosomes by Gram-negative bacteria, even when they encounter their tough outer membrane. The cubosomal formulations have been utilized in enhancing the antibacterial activity of some antibiotics, including gatifloxacin [33], erythromycin [34], and rifampicin [35].

Here, we aimed to develop celecoxib in cubosomal NPs and evaluate the influence of cubosomal formulation on the in vivo antibacterial efficiency of celecoxib against *S. aureus* through a systemic infection model in mice, using different histological, immunohistochemical, and molecular techniques. The cubosomal nanodispersion is expected to enhance the antibacterial efficacy of celecoxib and overcome the challenges associated with bacterial resistance, thereby providing a potential alternative to current antibiotics, which suffer from resistance.

## 2. Results and Discussion

### 2.1. Evaluation of the Prepared Celecoxib-Loaded Cubosomes

**Figure 1:**
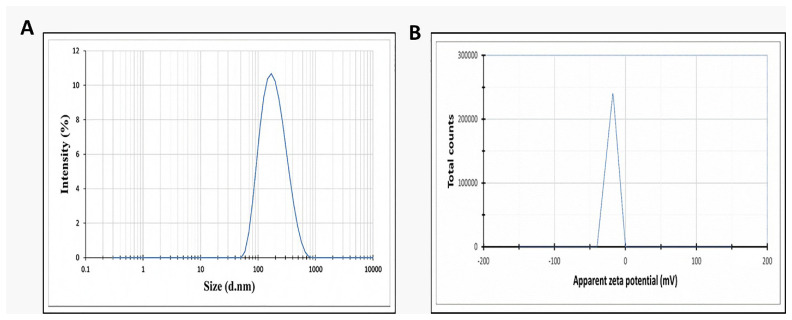
(**A**) Size distribution by intensity and (**B**) zeta distribution for the prepared celecoxib-loaded cubosomes.

The prepared celecoxib-loaded cubosomal NPs were assessed for particle size, surface charge, morphology, drug loading%, and EE%. The size distribution investigation revealed that the prepared celecoxib-loaded cubosomes had a mean particle size of 128.15 ± 3.04 nm with a low polydispersity index of 0.235 ± 0.023 (Figure 1A), which designates a narrow and homogenous size distribution of the prepared NPs. The zeta potential measurement revealed that the prepared NPs had a negative surface charge of −17.50 ± 0.45 (Figure 1B), indicating the formation of a highly stable nanodispersion with little susceptibility for particle aggregation. The presence of oleic acid in the GMO structure may be responsible for the resulting negative charge. The cubosomal dispersion exhibited an entrapment efficiency of 88.57 ± 2.36%. The higher value obtained for the EE% may result from the higher lipophilicity of celecoxib and the higher internal area of the cubosomal NPs. The drug loading% was also found to be 20.59 ± 1.9%.

**Figure 2:**
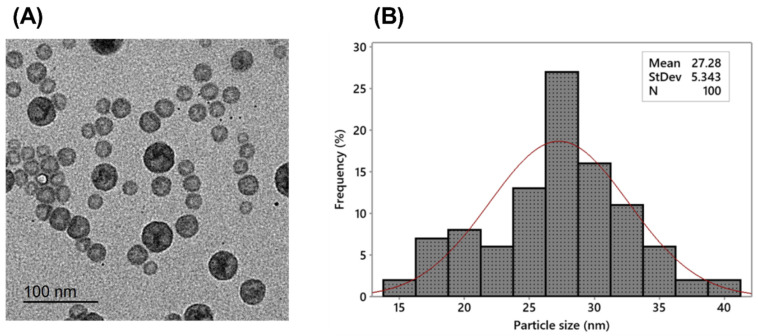
(**A**) Transmission electron photograph and (**B**) histogram (red line) of size distribution of the prepared celecoxib-loaded cubosomes.

The transmission electron micrograph (Figure 2A) of the prepared celecoxib-loaded cubosomes showed a narrow size distribution for the cubosomal NPs, which have a spherical shape and are non-aggregated; also, no disruption was detected even after applying mechanical forces such as probe sonication. After the size distribution analysis (Figure 2B), the mean particle size was found to be 27.28 ± 5.34 nm. It is essential to highlight that the hydrodynamic PS determined through dynamic light scattering (DLS) analysis was found to be larger compared to the size obtained from TEM micrographs. DLS is a technique that calculates the particle diameter based on multi-angle measurements in a liquid medium, whereas the TEM experiment provides the actual size of the particles in dry form [36].

### 2.2. In Vitro Release of the Celecoxib-Loaded Cubosomes

**Figure 3:**
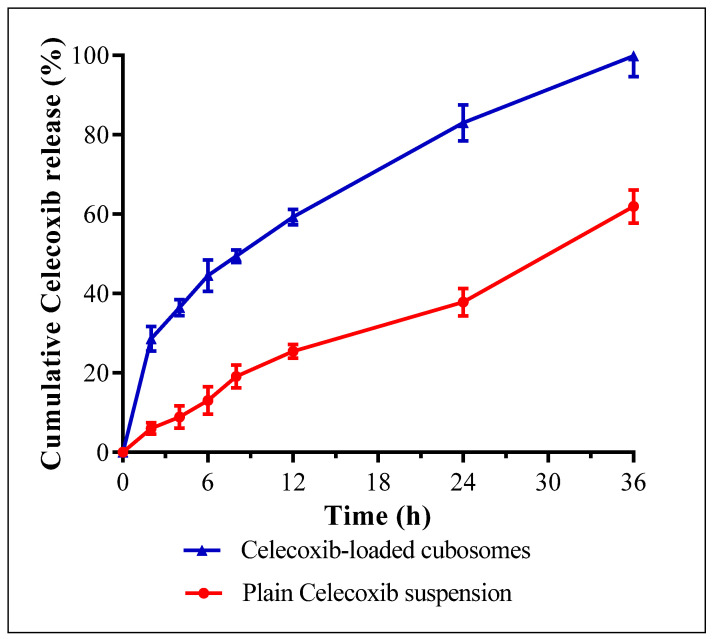
The mean cumulative release of plain celecoxib suspension and celecoxib-loaded cubosomes at pH 6.8.

The release profiles of the plain celecoxib suspension and celecoxib-loaded cubosomes are shown in Figure 3. The cubosomal dispersion exhibited a biphasic release profile with a faster initial release of 41.12 ± 3.68% after 6 h compared to 13.09 ±1.82% in the case of the plain celecoxib suspension. The initial burst release may be attributed to the unentrapped fraction and the adsorbed drug on the surface of the celecoxib-loaded cubosomes. The burst release of hydrophobic drugs from cubosomes was previously reported [28]. After 36 h, celecoxib-loaded cubosomal dispersion displayed a substantial (*p* ˂ 0.05) higher % cumulative celecoxib release (almost 100%) compared to 61.89 ± 4.16% for the plain celecoxib suspension. The higher drug release from the cubosomal dispersion may be due to the developed small particles in a nanometric range with a large surface area [37]. The kinetics analysis of the release data of the cubosomal dispersion was found to be the best fit to the Higuchi model with R^2^ of 0.997 ± 0.054, demonstrating that diffusion is the dominant mechanism of release. These findings agree with previous observations on the release of drugs from cubosomes [38,39,40].

### 2.3. In Vitro Antibacterial Activity

**Figure 4:**
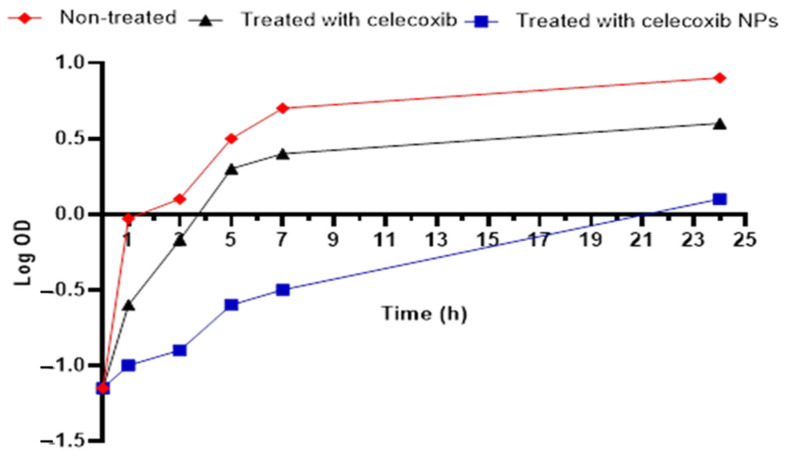
Growth curve of the studied isolate.

This test was performed as a primary investigation of the potential antibacterial action of celecoxib and its formulation against *S. aureus* in vitro. Interestingly, the celecoxib (free drug) and the celecoxib cubosomes revealed antibacterial activity, shown by the inhibition zones around the wells (Figure S1). Also, the minimum inhibitory concentration (MIC) of celecoxib and its cubosomal NPs were 256 µg/mL and 16 µg/mL, respectively. The growth curve of the studied isolate after treatment with celecoxib and its cubosomal NPs is revealed in Figure 4, as the NPs significantly delayed the growth of the studied isolate. In order to develop novel antimicrobials, a long time is required, and this will not allow us to cope with the fast spread of bacterial resistance worldwide. Thus, drug repurposing is evolving as a good idea for combating pathogenic bacteria [41]. In this regard, the cost and time are reduced compared to the conventional methods of antibiotic discovery [42]. Thus, in the current study, celecoxib was tested for its potential antibacterial activity in vitro. Also, to achieve the highest benefit from this drug as an antibacterial agent, it was formulated in cuposomal NPs to improve its in vivo oral bioavailability.

### 2.4. In Vivo Antibacterial Action

#### 2.4.1. Bacterial Load

**Figure 5:**
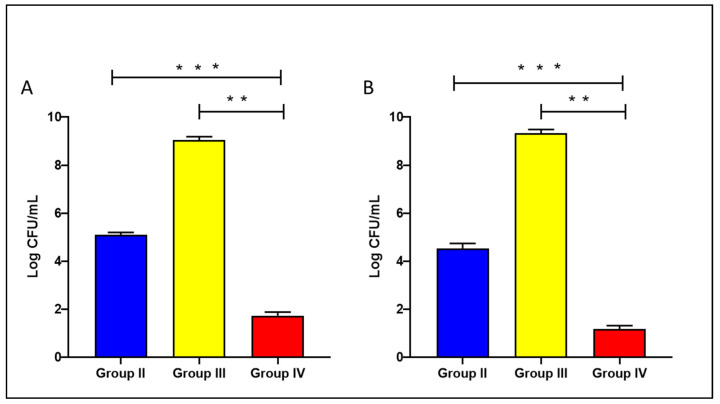
Bacterial load in the liver (**A**) and spleen (**B**) of the experimental groups. The double asterisks represent a substantial difference (*p* < 0.05) between groups III and IV. The triple asterisks denote a substantial difference (*p* < 0.05) between groups II and IV. Group II: plain celecoxib, group III: blank formula without celecoxib, and group IV: celecoxib cubosomal formulation.

The level of bacteria in the liver and spleen of the tested groups was assessed to reveal the impact of treatment with the celecoxib cubosomes on the bacterial count in the studied tissues, as shown in Figure 5. 

A study conducted by Annamanedi et al. [43] reported that using celecoxib with low doses of antibiotics significantly reduced the bacterial burden in various organs in an infected murine model with different bacterial types.

**Figure 6:**
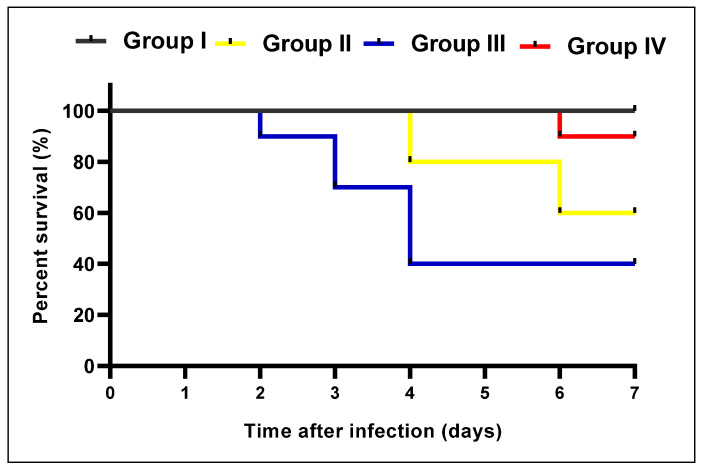
Kaplan–Meier survival curve. Group I: normal control, group II: plain celecoxib, group III: blank formula without celecoxib, and group IV: celecoxib cubosomal formulation.

The survival curve of the mice in the different experimental groups is shown in Figure 6.

#### 2.4.2. Histological Features 

**Figure 7:**
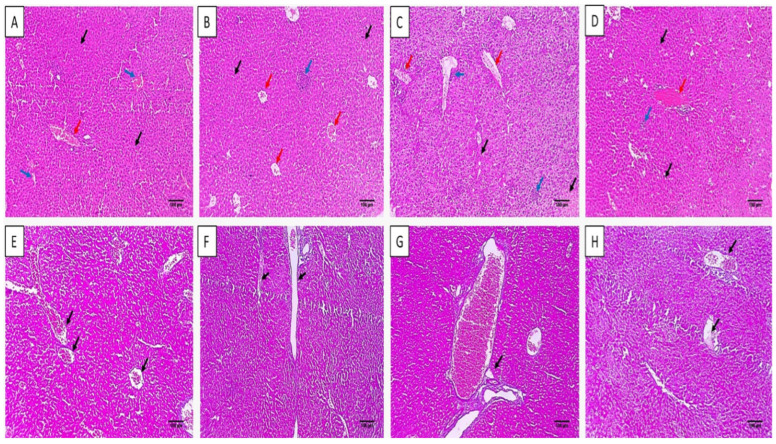
H&E staining of liver sections of (**A**) group I with central vein with an average-sized (blue arrows) portal tract (red arrow), bounded by hepatocytes cords, and isolated by blood sinusoids (black arrows) (×100). (**B**) Group II with central veins of an average size (red arrows) bounded by hepatocytes cords (black arrows) and focal inflammation (blue arrow) with no necrosis (×100). (**C**) Group III with congested central veins (red arrows) bounded by chronic inflammation (blue arrows) and hepatocytes displaying focal necrosis (black arrows) (×100). (**D**) Group IV with normal portal tract (red arrow) bounded by normal cords of hepatocytes (black arrows) and few inflammatory cells (blue arrow) with no necrosis (×100). Masson’s trichrome stain of liver sections of (**E**) group I with normal portal tract and central veins with a slight fibrous wall (black arrows) and absence of fibrosis (score 0) (×100). (**F**) Group II with fibrous development of certain portal areas and fibrous septa (black arrow) (score 2) (×100). (**G**) Group III with the fibrous expansion of all portal areas with portal–portal bridging (black arrows) (score 3) (×100). (**H**) Group IV with fibrous development of a few portal areas with an absence of fibrous septa (black arrow) (score 1) (×100).

**Figure 8:**
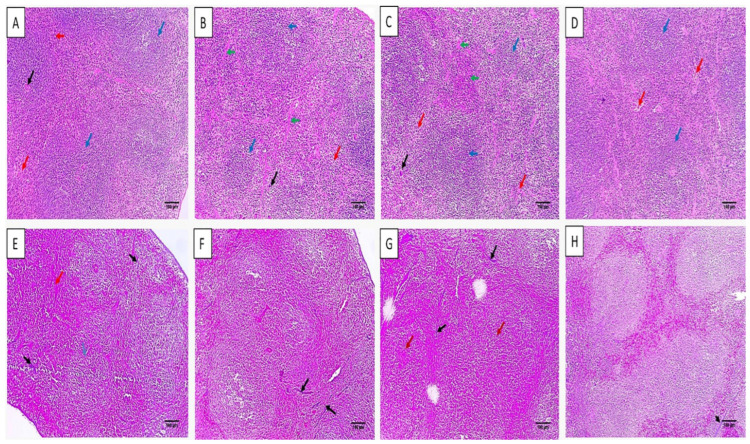
H&E staining of spleen sections of (**A**) group I with white pulp of normal size (lymphoid follicles) (blue arrows) and central arteriole (black arrow) bounded by red pulp of average size (blood sinusoids) (red arrows) (×100). (**B**) Group II with red pulps that have an average size (red arrows) with short bands of fibrosis (green arrows) bounded by a few atrophic white pulps (blue arrows) (×100). (**C**) Group III with significant congestion exhibiting mild congested red pulp (red arrows) with extramedullary hematopoiesis showing megakaryocytes (black arrow) with focal fibrosis (green arrows), bounded by some white pulp showing atrophy (blue arrows) (×100). (**D**) Group IV with normal-sized red pulps (red arrows) bounded by normal-sized white pulp (blue arrows) (×100). Masson’s trichrome stain of spleen sections of (**E**) group I with parenchyma formed of white (blue arrow) and red pulps (red arrow). The stroma was characterized by minor blue-stained collagen fibers (black arrows) (×100). (**F**) Group II with a mild decline of collagen fibers with residual streaks of blue-stained collagen fibers (black arrows) (×100). (**G**) Group III with significant congestion (red arrows), with some areas of blue-stained collagen fibers (black arrows) (×100). (**H**) Group IV with an obvious decline of the collagen fibers with few collagen streaks (black arrow) (×100).

The histological characters of the liver and spleen were revealed by staining with hematoxylin and eosin (H&E) stain, as shown in Figure 6 and Figure 7. Also, the collagen staining of the different experimental groups was studied using Masson’s trichrome stain (Figure 7 and Figure 8). 

H&E staining of liver and spleen sections of group I showed normal features of the central vein with an average-sized portal tract confined by hepatocytes cords and isolated by blood sinusoids. The spleen had a white pulp of normal size (lymphoid follicles), and the central arteriole was bounded by red pulp that had average size. Conversely, group III revealed focal inflammation in both liver and spleen tissues. The histological features were improved in group IV as there was a normal portal tract bounded by normal cords of hepatocytes and few inflammatory cells. Spleen tissues revealed normal-sized red pulps bounded by normal-sized white pulp.

Masson’s trichrome stain was used to investigate the fibrosis of liver and spleen sections. The group treated with celecoxib cubosomes revealed a noticeable decline of the collagen fibers compared to the normal group.

**Figure 9:**
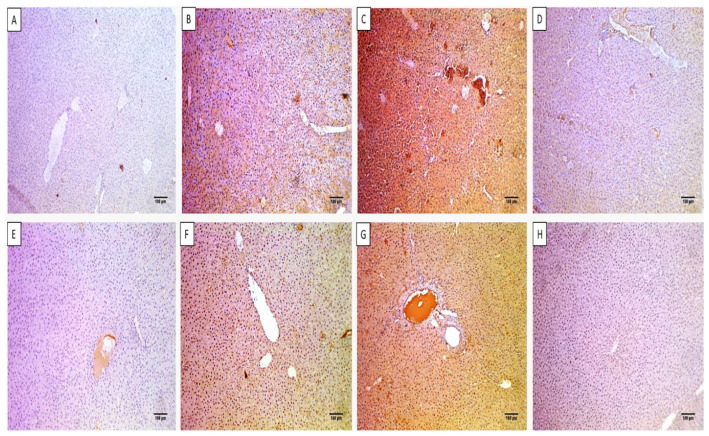
Immunohistochemical staining of the liver sections of (**A**) group I with a negative expression of caspase-3 (0.68%) (×100). (**B**) Group II with a moderate positive caspase-3 expression (45.76%) (×100). (**C**) Group III with a strong caspase-3-positive expression (73.32%) (×100). (**D**) Group IV with a negative caspase-3 expression (8.56%) (×100). (**E**) Group I with a negative expression of NF-kβ (0.74%) (×100). (**F**) Group II with a moderate expression of NF-kβ (48.28%) (×100). (**G**) Group III with a strong expression of NF-kβ (72.54%) (×100). (**H**) Group IV with a negative expression of NF-kβ (6.24%) (×100).

**Figure 10:**
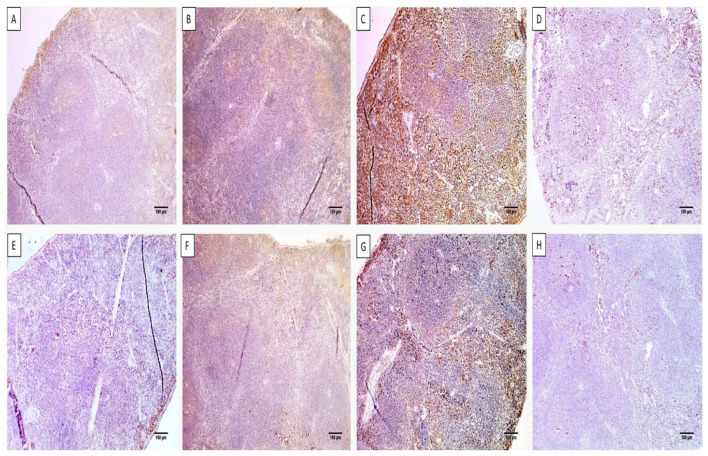
Immunohistochemical staining of the spleen sections of (**A**) group I with a negative expression of caspase-3 (5.23%) (×100). (**B**) Group II with a moderate positive caspase-3 expression (47.04%) (×100). (**C**) Group III with a strong caspase-3-positive expression (79.43%) (×100). (**D**) Group IV with a weak positive caspase-3 expression (17.43%) (×100). (**E**) Group I with a negative expression of NF-kβ (2.9%) (×100). (**F**) Group II with a moderate expression of NF-kβ (29.03%) (×100). (**G**) Group III with a strong expression of NF-kβ (57.23%) (×100). (**H**) Group IV with a negative expression of NF-kβ (4.54%) (×100).

The immunohistochemical studies elucidated the immunostaining of the liver and spleen tissues with caspase-3 and nuclear factor kappa β (NF-kβ), as shown in Figure 9 and Figure 10.

Apoptosis and inflammation usually accompany systemic infections. Thus, the drugs which inhibit both processes by inhibiting the enzymes and mediators involved in them are very beneficial in inhibiting the pathogenesis of bacterial infections [44]. Thus, improving the oral bioavailability of celecoxib by its formulation in cubosomes is useful in treating bacterial infection. 

Caspase-3 is an enzyme that is involved in apoptosis or programmed cell death [45], and it was found that the immune expression of this enzyme was substantially reduced in the livers and spleens of the celecoxib-cubosomes-treated group.

Regarding NF-kβ, it mediates the inflammatory process by induction of the release of the proinflammatory mediators [45,46]. Here, celecoxib cubosomes substantially reduced both markers, as revealed by immunostaining. 

#### 2.4.3. ELISA and qRT-PCR

**Figure 11:**
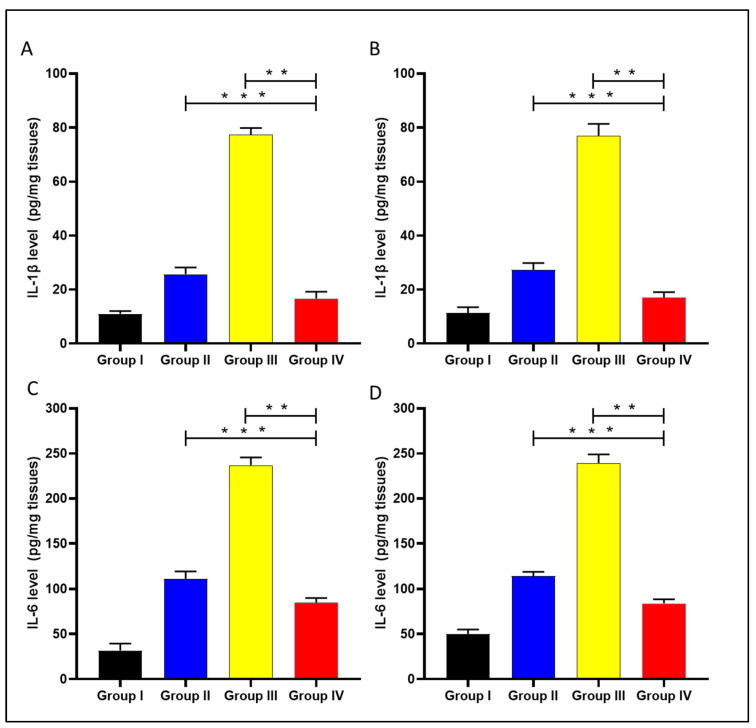
Graph presenting the level of IL-1β in the liver (**A**) and spleen (**B**) and showing the level of IL-6 in the liver (**C**) and spleen (**D**). The double asterisks represent a substantial difference (*p* < 0.05) between groups III and IV. The triple asterisks denote a considerable difference (*p* < 0.05) between groups II and IV. Group I: normal control, group II: plain celecoxib, group III: blank formula without celecoxib, and group IV: celecoxib cubosomal formulation.

The levels of the interleukin-1 beta (IL-1β) and interleukin-6 (IL-6) were determined in the liver and spleen of the experimental groups using ELISA, as displayed in Figure 11. 

**Figure 12:**
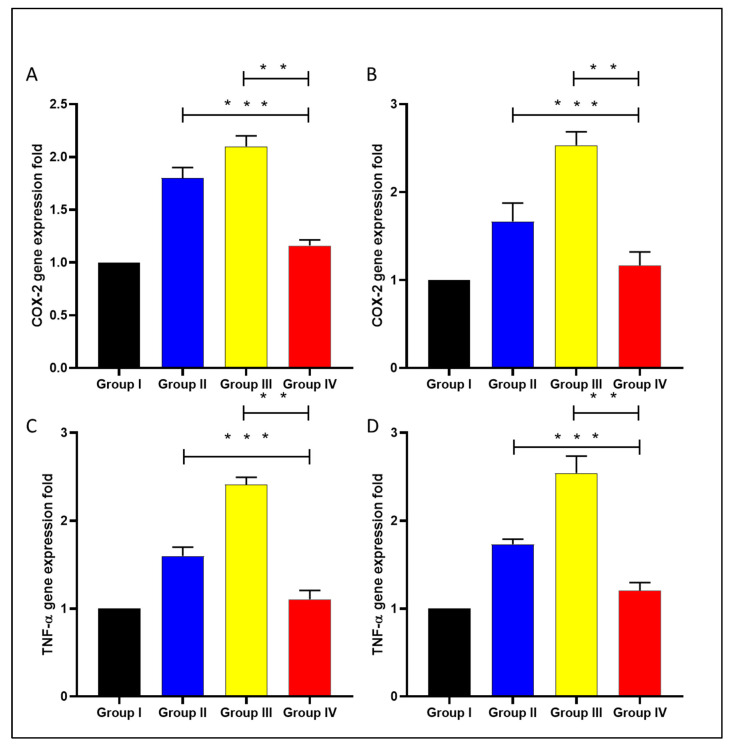
A chart revealing the fold change of COX-2 in the liver (**A**), spleen (**B**), and TNF-α fold change in (**C**) in the liver and spleen (**D**). Group I: normal control, group II: plain celecoxib, group III: blank formula without celecoxib, and group IV: celecoxib cubosomal formulation. The double asterisks represent a substantial difference (*p* < 0.05) between groups III and IV. The triple asterisks denote a considerable difference (*p* < 0.05) between groups II and IV.

Cyclooxygenase-2 (COX-2) and tumor necrosis factor-alpha (TNF-α) gene expression were analyzed in the liver and spleen of the experimental groups using qRT-PCR, as displayed in Figure 12.

Celecoxib is an anti-inflammatory drug that works by inhibiting COX-2 enzyme activity. It could be beneficial in treating bacterial infections, as inflammation usually accompanies viral and bacterial infections [47,48]. Here, celecoxib cubosomal NPs resulted in a substantial decrease (*p* < 0.05) in the inflammatory markers, including IL-1β, IL-6, COX-2, and TNF-α compared with celecoxib-free drugs. This could be explained by the cubosomal NPs enhancing celecoxib bioavailability. COX-2 inhibition was reported to improve the clearance of *Burkholderia pseudomallei* and *Pseudomonas aeruginosa* from in vivo models of infected mice [49]. Also, it was reported that the inflammatory markers, including IL-1β [50], IL-6 [51], and TNF-α [52], increase in bacterial infections, which participate in bacterial pathogenesis.

## 3. Materials and Methods

### 3.1. Chemicals

Celecoxib was obtained from Merck (Rahway, NJ, USA). Monoolein (GMO) was received as a gift from Kerry Ingredients & Flavours (Kerry, Zwijndrecht, The Netherlands). Poloxamer 407 (P 407), cellulose dialysis membrane (MWCO = 12,000 g/mole), and methanol (HPLC-grade) were obtained from Merck, (Rahway, NJ, USA).

### 3.2. Preparation and Characterization of Celecoxib-Loaded Cubosomes

Celecoxib-loaded cubosomal dispersions were formed, as previously explained [39]. Briefly, three grams of GMO was added to 0.3 g of P 407, and then melted in a water bath at 60 °C. An alcoholic solution of celecoxib was added to the molten mixture. The alcohol was then evaporated using a water bath. A transparent homogenous gel was obtained by adding the resulting mix to preheated deionized water (3 mL) under continuous agitation at 60 °C. The formed cubic gel was left for two days at ambient temperature for equilibration. A high-speed vortex was used to disperse the gel in deionized water. Finally, the dispersion was subjected to probe sonication for five min using a plus mode (five seconds on and three seconds off) to achieve the final celecoxib-loaded cubosomal NPs. The concentration of celecoxib in the dispersion was 10 mg/g. A blank cubosomal dispersion was prepared by the same procedure without adding celecoxib. Amber glass vials were used to store the cubosomal dispersion at 4 °C until usage.

Size dispersion and zeta potential of celecoxib-loaded cubosomes were explored using a Zetasizer Nano ZS (Malvern, UK) after appropriate dilution and analyzed in triplicate at 25 ± 0.5 °C [53].

Cubosomes’ morphology was assessed using a transmission electron microscope (TEM) (Jeol JemDos, Tokyo, Japan). A droplet of the suspension was adsorbed on a carbon-covered copper grid and then air-dried. Before analysis, samples were stained with a 1% sodium phosphor tungstate solution.

The entrapment efficiency (EE%) of the prepared celecoxib-loaded NPs was evaluated after separating the unentrapped free drug using the centrifugation ultrafiltration approach. Briefly, the prepared dispersion (1 mL) was diluted correctly, and then the diluted sample was mixed with methanol to dissolve cubosomes and achieve the total amount of celecoxib (Q_0_). The free unentrapped celecoxib amount (Q_1_) was calculated by centrifugal filtration of the diluted sample at 6000 rpm for 15 min. The amount of celecoxib in the attained supernatant was measured using a UV-visible spectrophotometer (Jenway, Berlin, Germany) at a maximum wavelength of 254 nm [54] using the supernatant of the blank cubosomal dispersion as blank. The calibration curve was created in methanol and was linear at drug concentrations ranging from 5 to 20 µg/mL with an R^2^ of 0.998. The EE% was calculated according to the following equation:

$$EE\%=\frac{Q_{0}-Q_{1}}{Q_{0}}\times 100$$

while the drug loading (DL%) was calculated by the following equation:

DL% = (Weight of drug in cubosomes/Total weight of cubosomal components) × 100

### 3.3. In Vitro Release Study

The sample was assessed using the dynamic dialysis technique [37]. A volume of drug-loaded NPs and plain celecoxib suspension equivalent to one milligram of celecoxib was put in a dialysis bag previously soaked overnight in phosphate-buffered saline (PBS) at pH 6.8 to ensure complete swelling of the membrane and to obtain pores with a fixed diameter. The dialysis bag was placed in PBS as a release medium (pH 6.8) with 1% sodium lauryl sulphate to keep sink conditions and magnetically stirred at 37 °C and 100 rpm. At different times (0, 2, 4, 6, 8, 12, 24, and 36 h), three milliliters of the samples were withdrawn and compensated by the same volume of fresh medium. The drug amount was detected at 254 nm spectrophotometrically.

### 3.4. Antibacterial Potential (In Vitro) 

#### 3.4.1. Agar Well Diffusion

The potential antibacterial action of celecoxib and its cubosomal NPs was investigated in vitro using the agar well diffusion method in Muller–Hinton agar plates, as reported previously [55,56]. *Staphylococcus aureus* ATCC 29,231 standard isolate was utilized in the current study. The plates were incubated for 24 h at 37 °C and inspected for inhibition zones around the wells containing the tested drugs.

#### 3.4.2. Determination of MIC Values

MIC values of celecoxib and its cubosomal NPs were determined by broth microdilution method [57]. Each plate had a positive control (bacterial suspension) and a negative control (broth). MIC values were recorded as the lowest concentration that resulted in complete growth inhibition.

#### 3.4.3. Growth Kinetics

The impact of celecoxib and its cubosomal NPs on the growth of the bacterial isolate was revealed at 0.5 MIC values, as previously reported [57]. The optical density (OD) of the *S. aureus* isolate was recorded at 620 nm using UV–Vis spectrophotometer (SHIMADZU, Kyoto, Japan) at different times (0, 1, 3, 5, 7, and 24 h). The growth curve was constructed by plotting log OD620 against time (h).

### 3.5. Experimental Protocol (In Vivo)

#### 3.5.1. Animals

Forty mice were acquired from the faculty of veterinary medicine at Cairo University, Cairo, Egypt. All standard conditions for feeding and preserving animals were followed as previously described [58]. The protocol of the in vivo study was approved by the research ethics committee of the faculty of pharmacy, Tanta University, Tanta, Egypt (TP/RE/5/23 p-0022).

#### 3.5.2. Experiment

The antibacterial efficiency of the celecoxib cubosomal formulation against *S. aureus* was explored in vivo. The bacterial suspension (10^7^ colony forming unit (CFU)/mL) was injected for two days [56] to induce infection in mice. With a random distribution, the animals were classified into the following groups: group I (normal control, not injected with bacteria and administered 0.9% saline, orally each day), group II (injected with bacteria and administered 5 mg/kg celecoxib, orally each day) [43], group III (injected with bacteria and administered 5 mg/kg blank formula without celecoxib, orally each day), and group IV (injected with bacteria and administered 5 mg/kg celecoxib cubosomal formulation, orally each day). After seven days, mice were anesthetized, and liver and spleen tissues were obtained. The tissue’s bacterial burden was determined by counting the CFU/mL in the tissue homogenate, and the survival rate was determined using Kaplan–Meier survival curve [59]. 

#### 3.5.3. Histological Studies

According to the previously reported method [56], the histological characters of the liver and spleen were studied using H&E stain as well as Masson’s trichrome stain. The scores of fibrosis were given as previously documented [60,61]. Regarding the immunohistochemical analysis of the studied tissues, caspase-3 and NF-kβ monoclonal antibodies were utilized for staining of the tissues, and they were examined by light microscope and given scores according to the percentage of the positive immune cells as previously described [62].

#### 3.5.4. ELISA

The levels of the inflammatory markers (IL-1β and IL-6) in pg/mg protein were determined using an ELISA kit (Abcam Co., Cambridge, UK).

#### 3.5.5. qRT-PCR

The relative gene expression of the COX-2 and TNF-α were determined in the liver and spleen tissues of the different tested groups. Total RNA was extracted using the Purelink™ RNA kit (Thermo Scientific, Waltham, MA, USA), and then converted into cDNA by the Power™ cDNA kit (iNtRON Biotechnology, Seongnam, Kyonggi-do, Republic of Korea) to synthesize the cDNA. Rotor-Gene Q 5plex (Qiagen, Hilden, Germany) was utilized in the qRT-PCR run, and the employed primers are revealed in Table S1.

### 3.6. Statistics

Data were presented as mean ± standard deviation, and ANOVA was employed to study the experimental group differences. The difference was regarded to be significant if *p* < 0.05. The software utilized was Prism version 8 (GraphPad Software, San Diego, CA, USA).

## 4. Conclusions

This work aimed to develop celecoxib-loaded cubosomes and evaluate the in vivo antibacterial action of the cubosomal formulation against *S. aureus*. In vitro, celecoxib-loaded cubosomes entrapped 88.57 ± 2.36% of the drug and demonstrated a spherical shape using TEM with a narrow nano-size distribution. Celecoxib cubosomes led to an enhancement of the in vivo antibacterial potential against *S. aureus* isolate in comparison with the celecoxib-free drug. This was revealed by improved histological features using H&E as well as Masson’s trichrome stains. Also, there was an improvement in the immunohistochemical features. The celecoxib-loaded formulation exhibited a significant decrease in the inflammatory biomarkers (IL-1β and IL-6) using ELISA. In addition, qRT-PCR was employed, and it revealed a downregulation of the genes encoding COX-2 and TNF-α in the celecoxib-formulation-treated group. Thus, to the best of our knowledge, this is the first study that presented cubosome nanovesicles as an optimized formula for the delivery of repurposed celecoxib for the treatment of systemic *S. aureus* infection. This study presents a solution to the increasing resistance of *S. aureus* bacteria. In future studies, the antibacterial action of celecoxib should be investigated on other resistant bacterial pathogens, alone and in combination with different antibiotics.

## Data Availability

All data generated and analyzed during this study are included in this article and its Supplementary Information files.

## Supplementary Materials

The following supporting information can be downloaded at: https://www.mdpi.com/article/10.3390/microorganisms11092247/s1, Figure S1. Inhibition zone of the studied formula. Table S1. Primer sequences [63,64,65].
