# Supplementary material for: Celecoxib-Loaded Cubosomal Nanoparticles as a Therapeutic Approach for Staphylococcus aureus In Vivo Infection
Source: Microorganisms. 2023 Sep 6;11(9):2247. doi: 10.3390/microorganisms11092247 (PMC10535980; doi:10.3390/microorganisms11092247)
Supplement: Supplementary file 1 [file microorganisms-11-02247-s001.zip › microorganisms-2569498-supplementary.pdf]

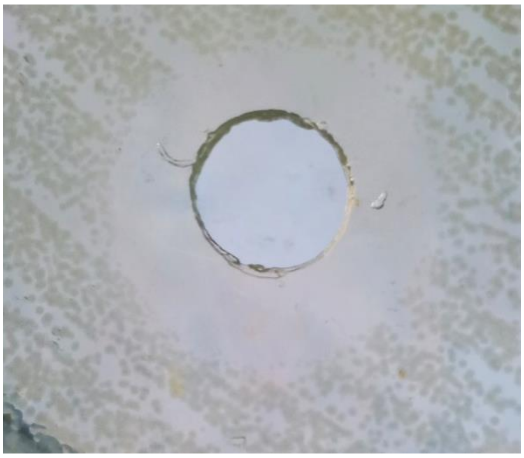

Figure S1. Inhibition zone of the studied formula.

Primer	Sequence	Reference
GAPDH	5′-AGAAGG CTGGGGCTCATTTG-3′<br>5′-AGGGGCCAT CCACAGTCTTC-3′	[1]
COX-2	5′-ACA CAC TCT ATC ACT GGC ACC-3′<br>5′-TTC AGG GAG AAG CGT TTG C-3′	[2]
TNF-α	5′-GCCTCTTCTCATTCCTGCTTG-3′<br>5′-CTGATGAGAGGGAGGCCATT-3′	[3]

Table S1. Primer sequences

1. Alotaibi, B.; El-Masry, T. A.; Elekhnawy, E.; El-Kadem, A. H.; Saleh, A.; Negm, W. A.; Abdelkader, D. H., Aqueous core epigallocatechin gallate PLGA nanocapsules: Characterization, antibacterial activity against uropathogens, and in vivo reno-protective effect in cisplatin induced nephrotoxicity. *Drug Delivery* **2022,** 29, (1), 1848-1862.
2. Fornai, M.; Blandizzi, C.; Colucci, R.; Antonioli, L.; Bernardini, N.; Segnani, C.; Baragatti, B.; Barogi, S.; Berti, P.; Spisni, R., Role of cyclooxygenases 1 and 2 in the modulation of neuromuscular functions in the distal colon of humans and mice. *Gut* **2005,** 54, (5), 608-616.
3. Yamakawa, I.; Kojima, H.; Terashima, T.; Katagi, M.; Oi, J.; Urabe, H.; Sanada, M.; Kawai, H.; Chan, L.; Yasuda, H., Inactivation of TNF-α ameliorates diabetic neuropathy in mice. *American Journal of Physiology-Endocrinology and Metabolism* **2011**.
